# Bacterial Pathogens in the Food Industry: Antibiotic Resistance and Virulence Factors of *Salmonella enterica* Strains Isolated from Food Chain Links

**DOI:** 10.3390/pathogens11111323

**Published:** 2022-11-10

**Authors:** Michał Wójcicki, Agnieszka Chmielarczyk, Olga Świder, Paulina Średnicka, Magdalena Strus, Tomasz Kasperski, Dziyana Shymialevich, Hanna Cieślak, Paulina Emanowicz, Monika Kowalczyk, Barbara Sokołowska, Edyta Juszczuk-Kubiak

**Affiliations:** 1 Laboratory of Biotechnology and Molecular Engineering, Department of Microbiology, Prof. Waclaw Dabrowski Institute of Agricultural and Food Biotechnology—State Research Institute, Rakowiecka 36 Street, 02-532 Warsaw, Poland; 2 Department of Microbiology, Faculty of Medicine, Jagiellonian University Medical College, Czysta 18 Street, 31-121 Cracow, Poland; 3 Department of Food Safety and Chemical Analysis, Prof. Waclaw Dabrowski Institute of Agricultural and Food Biotechnology—State Research Institute, Rakowiecka 36 Street, 02-532 Warsaw, Poland; 4 Culture Collection of Industrial Microorganisms—Microbiological Resources Center, Department of Microbiology, Prof. Waclaw Dabrowski Institute of Agricultural and Food Biotechnology—State Research Institute, Rakowiecka 36 Street, 02-532 Warsaw, Poland; 5 Department of Microbiology, Prof. Waclaw Dabrowski Institute of Agricultural and Food Biotechnology—State Research Institute, Rakowiecka 36 Street, 02-532 Warsaw, Poland

**Keywords:** *Salmonella*, foodborne pathogens, virulence factors, antibiotic resistance, food safety

## Abstract

*Salmonella* is one of the most important foodborne pathogens. Fifty-three strains of *Salmonella* deposited in the Culture Collection of Industrial Microorganisms—Microbiological Resources Center (IAFB) were identified using molecular and proteomic analyses. Moreover, the genetic similarity of the tested strains was determined using the PFGE method. Main virulence genes were identified, and phenotypical antibiotic susceptibility profiles and prevalence of resistance genes were analyzed. Subsequently, the occurrence of the main mechanisms of β-lactam resistance was determined. Virulence genes, *inv*A, *fim*A, and *stn* were identified in all tested strains. Phenotypic tests, including 28 antibiotics, showed that 50.9% of the strains were MDR. The *tet* genes associated with tetracyclines resistance were the most frequently identified genes. Concerning the genes associated with ESBL-producing *Salmonella*, no resistance to the TEM and CTX-M type was identified, and only two strains (KKP 1597 and KKP 1610) showed resistance to SHV. No strains exhibited AmpC-type resistance but for six *Salmonella* strains, the efflux-related resistance of PSE-1 was presented. The high number of resistant strains in combination with multiple ARGs in *Salmonella* indicates the possible overuse of antibiotics. Our results showed that it is necessary to monitor antimicrobial resistance profiles in all food chain links constantly and to implement a policy of proper antibiotic stewardship to contain or at least significantly limit the further acquisition of antibiotic resistance among *Salmonella* strains.

## 1. Introduction

*Salmonella* is a Gram-negative, facultatively anaerobic, non-spore-forming bacteria of the *Enterobacteriaceae* family [1,2,3], including only two species: *Salmonella enterica* and *Salmonella bongori* [2]. Despite reports of the isolation of a third species of *Salmonella* called *S. subterranea* [4], newly released analyses have suggested that it is ultimately assigned to a different cluster, and thus, it has been reclassified to the species *Atlantibacter subterranea* [5].

*S. enterica* has six subspecies, namely, *Salmonella enterica* subsp. *enterica*, *Salmonella enterica* subsp. *salamae*, *Salmonella enterica* subsp. *arizone*, *Salmonella enterica* subsp. *diarizone*, *Salmonella enterica* subsp. *houtenae*, and *Salmonella enterica* subsp. *indica* [6]. The vast majority (about 99%) of *Salmonella* strains that cause infections in humans or other warm-blooded animals belong to the species *S*. *enterica* [7], which due to the wide variety, has been divided into groups and serological types and currently includes 2659 serovars [8]. The main reservoir of *S*. *enterica* subsp. *enterica* are breeding animals such as poultry, pigs, and cattle [9]. In humans, *S*. Typhi is responsible for systematic infections and typhoid fever, whereas paratyphoid is caused by the *S*. *enterica* of the Paratyphi A, Paratyphi B, or Paratyphi C serovars [10]. Other serovars, such as *S*. Enteritidis or *S*. Typhimurium, both in humans and animals, are associated with non-typhoidal salmonellosis [11,12].

*Salmonella* is one of the main causes of food poisoning resulting from the consumption of contaminated food and water [2,13,14]. It has been estimated that *Salmonella* causes 115 million human infections and 370,000 deaths per year globally [8]. According to the European Union One Health 2020 Zoonoses Report published in December 2021 by the European Food Safety Authority (EFSA) and European Centre for Disease Prevention and Control (ECDC) [15], salmonellosis is the second most commonly reported foodborne gastrointestinal infection in humans after campylobacteriosis and is an important cause of foodborne outbreaks in European Union Member States (EU MS) and non-MS countries. According to the above report, in 2020, the EU had the lowest number of reported cases of salmonellosis since 2007. It was probably related to both the COVID-19 pandemic and the withdrawal of the United Kingdom from the EU structures. According to ECDC, in 2020, 52,701 cases of salmonellosis were confirmed in the EU Member States, which corresponds to an EU reporting rate of 13.7 per 100,000 popular. A similar trend in the occurrence of salmonellosis was observed in 2016–2020. *S*. Enteritidis, *S*. Typhimurium, monophasic *S*. Typhimurium (1,4,[5],12:i:–), *S*. Infantis, and *S*. Derby were the most frequently isolated *Salmonella* serovars from hospitalized patients. In total, 22 MS reported 694 foodborne outbreaks of *Salmonella* in 2020, which caused 3686 diseases, 812 hospitalizations, and 7 deaths. *Salmonella* caused nearly a quarter (22.5%) of all foodborne outbreaks in 2020. The cause of the foodborne salmonellosis epidemic with strong evidence was eggs and egg products, pork and products thereof, and bakery products. In 2021, Poland itself reported to the Rapid Alert System for Food and Feed (RASFF) Systems 176 cases of *Salmonella* in food and feed (in 2020: 89 notifications). In Poland, 8269 cases were recorded, including 7975 food poisonings caused by *Salmonella* (in 2020: 5468 cases, including 5300 food poisonings). However, it should be emphasized that in 2021, the number of cases of all infectious diseases, except for COVID-19, was lower than in previous years due to, inter alia, limited social contacts [16,17].

Antibiotic resistance (AR) has rapidly evolved in the last few decades to become one of the greatest public health threats of the XXI century nowadays. The widespread use of antibiotics, especially the broad-spectrum ones, has contributed to the development of specialist drug defense strategies by bacterial pathogens [18,19]. The mechanisms of AR are then disseminated in the environment, for example, through horizontal gene transfer (HGT) between bacteria and by lysogenic bacteriophages (temperate phages) [18,19,20]. The World Health Organization (WHO) notes that *Salmonella* is one of the microorganisms in which some resistant serovars have emerged, affecting the food chain [21]. According to Commission Implementing Decision, 2013/652/EU, which applied from 1 January 2014 until December 2020, monitoring of AMR in *Salmonella* was mandatory in the major domestically produced animal populations and their derived meat. Specific monitoring of extended-spectrum β-lactamases (ESBLs-), AmpC- and carbapenemase-producing *Salmonella* was also required [22]. The analysis of AMR in *Salmonella* isolates from hospitalized humans included dominant serovars corresponding to those found in animal species [22,23]. WHO is strengthening the capacities of national and regional laboratories in the surveillance of foodborne pathogens as well as promoting the integrated surveillance of antimicrobial resistance (AMR) of bacterial pathogens in the food chain [21].

Thus, considering the above, our research aimed to determine the antibiotic resistance profile of *Salmonella* strains isolated from different food chain links.

## 2. Materials and Methods

### 2.1. Taxonomic Identification of the Salmonella Strains

A total of 53 *Salmonell*a strains used in this study were originally isolated from different food chain links (i.e., animals and animal breeding rooms, food production lines, food products, and hospitalized patients). The strains have been isolated since the 1980s and deposited in the Culture Collection of Industrial Microorganisms—Microbiological Resources Center (IAFB). The belonging of the isolated strains to the *Salmonella* genus was confirmed by amplification of the *16S* rRNA gene region. Bacterial DNA was isolated using a commercial DNeasy PowerFood Microbial Kit (Qiagen, GmbH, Hilden, Germany) and amplified with 16S–F (5′–AGAGTTTGATCCTGGCTCAG–3′) and 16S–R (5′–ACGGCTACCTTGTTACGACT–3′) primers [24]. The PCR conditions for the gene amplification were as follows: 2 min of initial denaturation at 95 °C, followed by 35 amplification cycles of denaturation at 94 °C for 30 s, hybridization at 51 °C for 35 s, and extension step at 72 °C for 1 min, ending with a final extension period of 72 °C for 10 min (SimpliAmp™ Thermal Cycler, Applied Biosystems™, ThermoFisher Scientific, Waltham, MA, USA). The amplicons were separated by electrophoresis on 2% agarose gel containing the SimplySafe™ interfering compound (5 μL/100 mL; EURx, Gdansk, Poland). To estimate the size of the amplicons, 5 μL of a DNA Ladder in the range of 100–3000 bp was used (A&A Biotechnology, Gdansk, Poland). Electrophoresis was carried out at 110 V for 60 min using the Sub-Cell GT Horizontal Electrophoresis System (Bio–Rad, Madrid, Spain). The bands were visualized using the GeneFlash Network Bio Imaging System (Syngene, Wales, UK). Sequencing was outsourced to Genomed S.A. company (Poland). Raw sequences were analyzed using BLASTn (NCBI) and deposited in the GenBank database. Moreover, taxonomic identification of bacterial strains was performed using proteomic profiles generated by MALDI–TOF–MS (Matrix-Assisted Laser Desorption Ionization Time-of-Flight Mass Spectrometry) analysis (Shimadzu Biotech, Manchester, UK).

### 2.2. Subtyping Salmonella Strains Using Pulsed-Field Gel Electrophoresis (PFGE)

PFGE was performed according to the international PulseNet CDC guidelines [25] and using the *Xba*I restriction enzyme. PFGE was performed using a CHEF DR–III PFGE system (Bio–Rad Laboratories, Inc., Hercules, CA, USA), and the following parameters were applied: separation on a 1% agarose gel (Pulsed Field Certified Agarose, Bio–Rad) in 0.5 M Tris–Borate–EDTA (TBE) buffer at 14 °C for 20 h (pulse times of 2.2–63.8 s). The gels were stained with 0.5 μg/mL of ethidium bromide for 15 min and photographed under UV transillumination using a QuantityOne (BioRad, Madrid, Spain) software and GelDoc 2000 (BioRad, Madrid, Spain) system. The banding patterns were analyzed with bionumerics Gel Compar II 6.5 software (Applied Maths, Sint–Martens–Latem, Belgium) using the Dice coefficient and the UPGMA (Unweighted Pair-Group Method with Arithmetic mean) algorithm. A position tolerance of 1% was adopted for the generation of a dendrogram. *Salmonella* strains with more than 95% similarity were clustered together as identical.

### 2.3. Detection of Virulence Genes in Salmonella Strains

*Salmonella* strains were tested for six virulent genes (*inv*A, *fim*A, *stn*, *spv*C, *spv*R, and *rck*) using PCR with sets of specific primer pairs (Table 1). Detailed parameters of individual PCR reactions are presented in Table S1 (Supplementary Materials). Amplicons were separated by electrophoresis, as described in Section 2.1. To estimate the size of the amplicons, a DNA Ladder in the range of 100–1000 bp was used (A&A Biotechnology, Gdansk, Poland).

### 2.4. Antimicrobial Sensitivity Testing

*Salmonella* strains were tested in vitro for their susceptibility to 28 antimicrobial agents (Oxoid, Hampshire, United Kingdom). Antimicrobial susceptibility tests were performed using a Kirby–Bauer disk diffusion method according to the European Committee on Antimicrobial Susceptibility Testing (EUCAST) [32] and Clinical and Laboratory Standards Institute (CLSI) [33] standards on Mueller–Hinton agar (Merck). The plates were incubated at 37 °C for 18 ± 2 h. The following antimicrobial agents belonging to eight different classes were tested: (1) penicillins: ampicillin (AMP, 10 μg), sulbactam/ampicillin (SAM, 20 μg), amoxicillin/clavulanic acid (AMC, 30 μg), piperacillin (PRL, 30 μg), piperacillin/tazobactam (TZP, 36 μg), ticarcillin/clavulanic acid (TTC, 85 μg); (2) cephalosporins: cefepime (FEP, 30 μg), cefotaxime (CTX, 5 μg), ceftaroline (CPT, 5 μg), ceftazidime (CAZ, 10 μg), ceftazidime/avibactam (CZA, 14 μg), ceftolozane/tazobactam (CT, 40 μg), ceftriaxone (CRO, 30 μg); (3) carbapenems: ertapenem (ETP, 10 μg), imipenem (IMP, 10 μg), meropenem (MEM, 10 μg); (4) monobactams: aztreonam (ATM, 30 μg); (5) fluoroquinolones: ciprofloxacin (CIP, 5 μg), pefloxacin (PEF, 5 μg), levofloxacin (LEV, 5 μg), moxifloxacin (MXF, 5 μg), ofloxacin (OFX, 5 μg), norfloxacin (NOR, 10 μg); (6) aminoglycosides: amikacin (AK, 30 μg), gentamycin (CN, 10 μg), tobramycin (TOB, 10 μg); (7) phenicols: chloramphenicol (C, 30 μg), and (8) sulfonamides: sulphamethoxazole/trimethoprim (SXT, 25 μg). The tests were made in triplicate, and the mean diameter of the inhibitory zones was calculated. Susceptibility of the isolates to antimicrobial agents was categorized (as susceptible or resistant) by measurement of the inhibition zone, according to interpretive criteria that adhered to the EUCAST guidelines. *Escherichia coli* ATCC 25922 was used as the reference strain. *Salmonella* strains resistant to three or more different antimicrobial classes were categorized as multidrug-resistant (MDR) isolates.

Multiple antibiotic resistance (MAR) phenotypes were recorded for *Salmonella* strains showing resistance to more than two antibiotics, and the MAR index [34] was calculated as:

(1) $$\mathrm{MAR}=\frac{\mathrm{Number}\;\mathrm{of}\;\mathrm{resistance}\;\mathrm{to}\;\mathrm{antibiotics}}{\mathrm{Total}\;\mathrm{number}\;\mathrm{of}\;\mathrm{antibiotics}\;\mathrm{tested}}$$

### 2.5. Determination of Antibiotics Resistance Profile of Salmonella Strains

Mueller–Hinton agar was used to culture the *Salmonella* strains overnight at 37 °C. Bacterial DNA was isolated using a commercial DNeasy PowerFood Microbial Kit (Qi-agen, GmbH, Hilden, Germany). The presence of twenty-five resistance genes (*str*A/*str*B, *aad*A, *aad*B, *aac*C, *flo*F, *flo*R, *cat*1, *cat*2, *mcr*1, *mcr*2, *mcr*3, *mcr*4, *mcr*5, *aph*AI-IAB, *aph*A1, *aph*A2, *tet*A, *tet*B, *tet*C, *sul*1, *sul*2, *sul*3, *dfr*A1, *dfr*A10, and *dfr*A12) were analyzed using specific primer pairs by conventional PCR reaction. The primer pairs sequences and PCR product size are shown in Table 2. Detailed parameters of individual PCR reactions are presented in Table S1 (Supplementary Materials). Amplicons were separated by electrophoresis, as described in Section 2.1. To estimate the size of the amplicons, a DNA Ladder in the range of 100–1000 bp or 100–3000 bp was used (A&A Biotechnology, Gdansk, Poland). *Escherichia coli* ATCC 25922 was used as the negative control.

### 2.6. Screening for Phenotypic and Genotypic Detection of β-lactamases-Producing Salmonella Strains

In the last stage of the research, the phenotypic and genotypic assessment of the ability to produce β-lactamases by *Salmonella* strains was carried out. Phenotypic detection of ESBL-producing *Salmonella* was performed by the double-disc synergy test (DDST) on Mueller–Hinton agar (Merck) with amoxicillin/clavulanic acid (AMC, 30 μg), cefepime (FEP, 30 μg), cefotaxime (CTX, 30 μg), and ceftazidime (CAZ, 30 μg) disks (Oxoid, Hampshire, UK). Samples were considered to be ESBL-positive when the inhibition zone around cefotaxime or ceftazidime increased toward the central disk with AMC [42]. Moreover, for the detection of ESBL- and carbapenemases-producing *Salmonella*, commercial selective media were used: CHROMagar ESBL and CHROMagar mSuperCARBA, respectively (Graso Biotech, Starogard Gdanski, Poland).

The presence of five *bla* genes (*bla*_TEM_, *bla*_CTX-M_, *bla*_SHV_, *bla*_CMY-2_, and *bla*_PSE-1_) related to resistance to β-lactams were analyzed using specific primer pairs by conventional PCR reaction. The primer pairs sequences and predicted PCR product size are shown in Table 3. Detailed parameters of individual PCR reactions are presented in Table S1 (Supplementary Materials). Amplicons were separated by electrophoresis, as described in Section 2.1. To estimate the size of the amplicons, a DNA Ladder in the range of 100–1000 bp was used (A&A Biotechnology, Gdansk, Poland). *Escherichia coli* ATCC 25922 was used as the negative control.

## 3. Results and Discussion

### 3.1. Source of Isolation and Taxonomic Identification of the Salmonella Strains

*Salmonella* strains deposited in the Culture Collection of Industrial Microorganisms—Microbiological Resources Center (IAFB) were used in this study. Strains were classified into the *Salmonella* genus based on biochemical features. A panel of 53 strains isolated from different food chain links: animals and animal breeding rooms (ABR, *n* = 9), food production lines (FPL, *n* = 3), food products (FP, *n* = 38), and hospitalized patients (HP, *n* = 3) was analyzed. The taxonomic affiliation of all strains to the genus *Salmonella* was confirmed either by molecular methods (amplification of the *16S* rRNA gene region) or by the analysis of proteomic profiles (using MALDI–TOF–MS). All nucleotide sequences of the strains have been deposited in the GenBank database (Table 4).

Genetic identification (*16S* rRNA amplification) of most *Salmonella* strains coincided with proteomic identification. For three strains, the identification with the use of the MALDI–TOF–MS allowed us to obtain the result of belonging to the genus of bacterial isolates. These three *Salmonella* strains (KKP 1002, KKP 1006, and KKP 1041) were isolated from food products (specific origin unknown). During the heat treatment of food, bacterial cells could be damaged, which could affect the identification result based on protein profiles.

### 3.2. Subtyping Salmonella Strains Using Pulsed-Field Gel Electrophoresis (PFGE)

**Figure 1:**
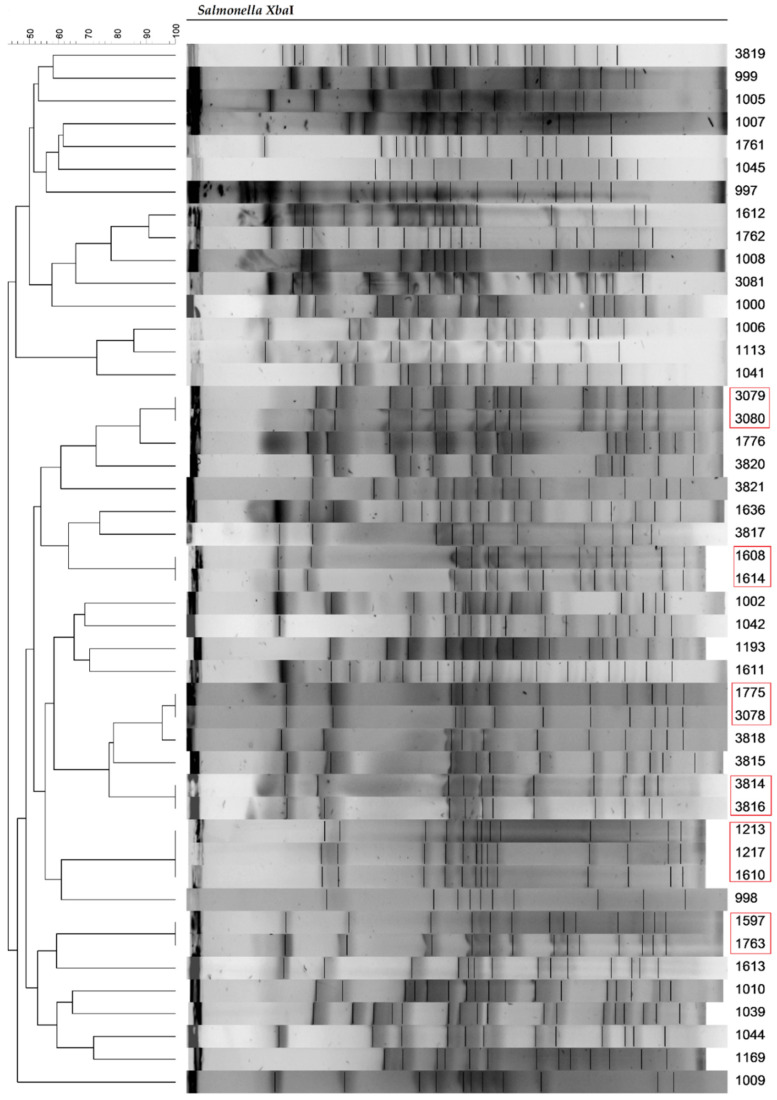
Dendrogram displaying PFGE profiles of *Salmonella* strains.

Pulsed-field gel electrophoresis (PFGE) was used to assess the genetic similarity of the *Salmonella* strains. For 7 *Salmonella* strains, including KKP 996, KKP 1001, KKP 1003, KKP 1004, KKP 1040, KKP 1043, and KKP 1514, the restriction pattern in PFGE was not obtained. Isolates that clustered >95% were considered the same clones (Figure 1). Genotyping of *Salmonella* strains by PFGE showed a relatively high diversity of isolates. Only a few tested strains had the same restriction pattern. Strains with identical restriction patterns are marked in red boxes (Figure 1).

### 3.3. Detection of Virulence Genes in Salmonella Strains

*Salmonella* encodes numerous genes such as *inv*A, *fim*A*, stn*, *spv*C*, spv*R*,* and *rck* involved in bacterial pathogenicity (Table 5) [47]. In our study, the presence of *inv*A gene in all tested *Salmonella* strains was confirmed. *inv*A located on pathogenicity island 1 (SPI-1, *Salmonella* Pathogenicity Islands 1) has been extensively studied for its ability to promote the virulence of *Salmonella* [47,48]. SPI-1 is required to invade host intestinal epithelium cells (the *inv*A gene is involved in this process) [49], induce an inflammatory reaction, and disrupt the host’s epithelial barrier [19,50]. The *fim*A and *stn* genes were also present in all tested strains. The *fim*A gene encodes the FimA protein, which is necessary for the assembly of type I fimbriae in *Salmonella* [51,52]. The fimbriae are *Salmonella* filamentous surface structures that contribute to the colonization of the host’s epithelium cells [47]. The *stn* gene encodes *Salmonella* enterotoxin, mainly associated with *S*. Typhi, *S*. Typhimurium, and *S*. Enteritidis serovar infections [53]. Clinically, the *stn* gene is a biomarker differentiating enterotoxic *S. enterica* strains from most *S. bongori* strains and other rods from the *Enterobacteriaceae* family [47,53,54]. In *Salmonella* strains, the *stn* gene exhibits high nucleotide sequence homology but limited similarity to its corresponding gene in other closely related enteric bacteria. Detection of the *stn* gene has been reported to be effective in detecting more than 50 strains of *S. enterica* and two strains of *S. bongori* without cross-reactivity to other more common intestinal strains [54]. The presence of the *spv*C and *spv*R genes was confirmed in 13 (24.5%) tested *Salmonella* strains. Moreover, in these strains, sequence of the *rck* gene was also detected. The *spv*C gene, present in plasmids and/or chromosomes, enhances the systemic proliferation of the bacterial pathogen and contributes to its replication outside the small intestine. Together with the *inv*A and *sse*L (located on the SPI-2), *spv*C facilitates the prediction of the overall pathogenicity, invasiveness, and replication potential of *Salmonella* [55]. The *spv*R gene product—SpvR is a regulator of the *spv*ABCD system, which is essential for systemic virulence [47]. The *spv* gene also encoded resistance to macrophage damage while the plasmid-borne Rck outer membrane protein (product of *rck* gene) confers resistance to complement killing [56]. In addition, The Rck protein has the ability to promote bacterial invasion of mammalian cells [57]. The expression of the *rck* gene is regulated by SdiA, a quorum sensing (QS) regulator, which is activated by acyl homoserine lactones (AHL) produced by other bacteria strains [58]. In our study, the presence of the *rck* gene was found in 20 (37.7%) tested *Salmonella* strains.

The presence of virulence genes in the *Salmonella* genome has been studied by many research groups, but the results are inconsistent. The *inv*A gene was present in all tested *Salmonella* strains, according to some studies [56,59]. Other authors reported that the *inv*A gene was present in 66% [47] and 91% [60] of the tested strains. A *Salmonella* virulence genes profile similar to the results obtained by our team was reported by Deguenon et al. [61], who confirmed the presence of the *inv*A, *fim*A, and *stn* in all *Salmonella* strains, while the *spv*C and *spv*R sequences were found in only 10% and 20% of the tested strains, respectively. In turn, Bolton et al. [56] determined the prevalence of the *rck* gene in *Salmonella* at the level of 62.1% (18/29). In other studies [62], including ESBL-producing *Salmonella*, the presence of the *rck* gene was not confirmed in any of the strains.

### 3.4. Antibiotic Resistance Profiles in Salmonella Strains

Antibiotics are usually used in the treatment of infections of bacterial etiology, and their widespread use in recent decades has led to a huge problem related to the antibiotic resistance of bacterial pathogens [63,64,65,66]. β-lactam antibiotics constitute the most numerous and most frequently used group of antibiotics [67,68]. This group includes four main subgroups: penicillins, cephalosporins, carbapenems, and monobactams [69]. The mechanism of action of β-lactams consists in interfering with the synthesis of the cell wall and inhibiting the formation of bridges connecting the peptidoglycan subunits. β-lactam antibiotics block the activity of the enzymes, including transpeptidases and carboxypeptidases, which are involved in the synthesis of peptidoglycan in the bacterial cell wall [68,70,71]. Fluoroquinolones (fluorinated quinolones, FQ) are commonly used in salmonellosis therapy [72,73], and their activity is associated with the inhibition of DNA synthesis by blocking topoisomerases II, DNA gyrase, and topoisomerase IV [74,75,76]. Another group of antibiotics used in the treatment of salmonellosis is aminoglycosides that bind to the 30S ribosome subunit, which leads to a disturbance in the reading of genetic information and inhibition of bacterial protein synthesis [77,78]. The mechanism of phenicol action also consists in inhibiting the synthesis of bacterial proteins but as a result of binding to the large (50S) ribosome subunit [79,80]. The last group of antibiotics tested in our study was sulfonamides. Sulfonamides are structural analogs of para-aminobenzoic acid (PABA) that inhibit the synthesis of folic acid and, indirectly, nucleic acids in bacterial cells [81,82,83].

In our study, *Salmonella* strains were tested for susceptibility to twenty-eight antimicrobial agents belonging to eight different classes (Table 6). Among the tested strains, seven (13.2%) showed no phenotype resistance to any of the tested antibiotics. All strains were sensitive to meropenem (carbapenem) and levofloxacin (fluoroquinolone). In this study, most of the *Salmonella* strains showed a MAR (Multiple Antibiotic Resistance) index lower than 0.3, whereas one of the strains (*S. enterica* strain KKP 998) showed a MAR index above 0.5 (MAR index = 0.61).

Moreover, a high prevalence of MAR was observed amongst the strains; 50.9% (27/53) of the isolates were MDR (Multi-Drug Resistant). *Salmonella enterica* strain KKP 998 (isolated from food product) exhibited the most extensive resistance profile to 17 antibiotics (AMC-TTC-FEP-CTX-CPT-CAZ-CT-CRO-ETP-IMP-ATM-PEF-MXF-OFX-AK -CN-TOB), belonging to 6 different classes of antibiotics (penicillins, cephalosporins, carbapenems, monobactams, fluoroquinolones, and aminoglycosides). Extensive resistance profiles were also exhibited by *S. enterica* strains KKP 3821, KKP 1004, KKP 1044, and KKP 3080. *S. enterica* strain KKP 3281 (isolated from animal breeding rooms) was resistant to 12 antimicrobials (FEP-CTX-CPT-CAZ-CZA-CT-CRO-ETP-ATM-PEF-AK-TOB) from 5 different classes of antibiotics (cephalosporins, carbapenems, monobactams, fluoroquinolones, and aminoglycosides), while the remaining three strains (isolated from food products) showed resistance to the 11 tested antibiotics. Some antibiotics were completely ineffective against tested bacteria (unpublished data). *S. enterica* strains KKP 1000 and KKP 3820 showed full growth with ampicillin, piperacillin, and chloramphenicol discs. Discs with sulphamethoxazole/trimethoprim (cotrimoxazole) did not inhibit the growth of *S. enterica* strains KKP 1007 and KKP 1010. In the case of *S. enterica* strain, KKP 3821 zones of growth inhibition were observed for five antibiotics (cefotaxime, ceftazidime, ceftazidime/avibactam, ceftolozane/tazobactam, and aztreonam). Moreover, as many as 14 strains of *Salmonella* (26.4%) were resistant to all tested antibiotics from the aminoglycosides class (i.e., amikacin, gentamycin, and tobramycin) (Table 6).

*Salmonella* strains showed the highest resistance to antibiotics from the aminoglycoside class (Table 7). Against amikacin, gentamicin, and tobramycin, phenotypic resistance was exhibited by 31 (58.5%), 26 (49.1%), and 24 (45.3%) strains, respectively. Ceftaroline, belonging to the class of broad-spectrum cephalosporins, was effective against the smallest number of strains tested. Thirty-two of the tested *Salmonella* strains (60.4%) were resistant to this antibiotic.

In our studies, we determined the sensitivity profiles of *Salmonella* strains and found a high percentage of strains exhibiting at least one phenotypic resistance. Some antibiotics from the penicillin class, macrolides or lincosamides were not used in the study due to a natural lack of activity against *Salmonella* [7]. The obtained results of antibiotic resistance indicate that *Salmonella* strains, isolated from different links of the food chain, are in a large percentage of MDR strains, i.e., they are insensitive to at least one antibiotic from at least three groups of antibacterial drugs used in the treatment of infections caused by *Salmonella* [84]. The results of studies published by Pławińska-Czarnak et al. [7] also confirm a high percentage (53.8%) of MDR *Salmonella* strains that showed resistance to β-lactams, aminoglycosides, cephalosporins, fluoroquinolones, sulfonamides, and tetracyclines. The high resistance to fifth-generation cephalosporins (ceftaroline), which are used in the treatment of severe bacterial infections, seems to be of concern. Among the tested *Salmonella* strains, as many as 60.4% were resistant to ceftaroline. Compared to the early-generation cephalosporins, ceftaroline has better stability to β-lactamases. However, it is inactivated by several classes of these enzymes and, thus, is not recommended for the treatment of ESBL-positive Gram-negative bacteria infections, as well as infections caused by bacteria producing metallo-β-lactamases or AmpC-type cephalosporinases [85]. The presence of a high percentage of strains resistant to the fifth generation of cephalosporins is an alarming situation, given the risk of transferring resistance genes in the environment. Ceftaroline is the drug of choice among cephalosporins and is active against multidrug-resistant *Staphylococcus aureus*, including MRSA, VRSA, and VISA [85,86]. Another class of antibiotics used in severe *Salmonella* infections is the sulfonamides; however, in this case, only 3.8% of the strains showed resistance. There was also no high percentage of strains resistant to carbapenems, which are used if ciprofloxacin and third-generation cephalosporin fail. In the study by Marin et al. [87], all isolated *Salmonella* strains showed resistance to at least one antibiotic, and 72% were MDR strains, with gentamicin–colistin and gentamicin–colistin–ampicillin being the most frequently observed resistance patterns. In a study conducted in China [88], 50.4% of the *Salmonella* isolates mostly originated from food products that were MDR. In total, 73% of the MDR *Salmonella* strains were resistant to tetracycline, 67% to ampicillin, and 59% to doxycycline. Our research shows a similar share of multidrug-resistant *Salmonella* strains (50.9%); however, significantly fewer of them were resistant to ampicillin (3.8%). Results obtained in another study carried out in Brazil [42] indicated that the highest percentage of *Salmonella* strains originated from broiler processing plants that were resistant to nalidixic acid and tetracycline. Strains resistant to meropenem, imipenem, and ciprofloxacin were not detected, while resistance to imipenem and ciprofloxacin was observed in 5.7% and 11.3% of *Salmonella* strains, respectively.

According to the latest report released by EFSA and ECDC [22], in the years 2019–2020 in the UE, there was a high percentage of *Salmonella* resistant to ampicillin, sulfonamides, and tetracyclines isolated from hospitalized patients. Zoonotic isolates showed moderate to very high resistance to these antibiotics. A very high percentage of FQ-resistant strains was observed in zoonotic isolates. *Salmonella* isolates from patients showed moderate resistance to ciprofloxacin. High resistance to third-generation cephalosporins has been observed neither for zoonotic strains nor those isolated from patients. In our study, none of the strains originated from hospitalized patients showed resistance to ampicillin and sulfonamides, but *S. enterica* KKP 996 and KKP 1193 strains (66% of strains isolated from hospitalized patients) showed genotypic resistance to tetracyclines (Table 8). Low percentage of FQ-resistant strains was observed amongst zoonotic isolates. Similar to the data collected in the EFSA/ECDC report, resistance to cefotaxime, ceftriaxone, and ceftazidime did not occur frequently (7.6%, 26.4%, and 5.7%, respectively) (Table 7). According to the EFSA/ECDC report, 25.4% of the strains isolated from patients were multidrug resistant. A significantly higher percentage of MDR strains was observed in *Salmonella* strains isolated from animals: 53.6% from broiler carcasses, 43.3% from pigs, and 23.1% from calves [22]. The above report [22] indicates the main etiological factors of *Salmonella* infections and underlines that special caution should be exercised regarding contact with raw materials and food of animal origin. Our outcomes confirmed that food is a common source of multidrug-resistant pathogenic bacteria (47.4% (18/38) MDR strains from food products and 55.6% (5/9) MDR strains from animals or animal breeding rooms).

### 3.5. Genotypic Resistance Profiles in Salmonella Strains

A genotypic resistance profile was determined for a panel of *Salmonella* strains using 25 primer pairs. *Salmonella* strains belonging to one clone in PFGE (Figure 1) did not show the same virulence profiles. The *aad*B and *aac*C genes encoding resistance to gentamicin (an aminoglycoside antibiotic) were not identified in any of the strains. There was also no presence of *mcr*1, *mcr*2, *mcr*3, *mcr*4, and *mcr*5 genes, encoding resistance to colistin, belonging to peptide antibiotics, and *dfr*A1, *dfr*A10 and *dfr*A12 genes associated with resistance to trimethoprim (dihydrofolic acid reductase inhibitor). Regarding the genes encoding chloramphenicol resistance, *cat*1 and *cat*2 were not found in any of the tested strains. However, the presence of the third chloramphenicol resistance gene (*flo*R) was confirmed in 7 (13.2%) of the tested strains. Phenotypic resistance to chloramphenicol was confirmed only in three *Salmonella* strains—KKP 999, KKP 1000, and KKP 3820 (Table 8). Among the two tested genes of resistance to neomycin (aminoglycoside antibiotic), the *aph*A1 was present in only one (1.9%) *Salmonella* strain (KKP 998), whereas *aph*A2 was not detected in any of the strains. In turn, the genes encoding resistance to sulfamethoxazole were also tested in the *Salmonella* strains, and out of the three tested genes (*sul*1, *sul*2, and *sul*3), no *sul*3 gene was found in any of the strains. Moreover, in seven *Salmonella* strains (i.e., KKP 1003, KKP 1040, KKP 1113, KKP 1611, KKP 1775, KKP 3816, and 3821), none of the tested resistance genes was identified. Importantly, only *S. enterica* strain KKP 1040 showed phenotypical sensitivity to all tested antibiotics with the simultaneous absence of all tested resistance genes.

The highest percentage of resistant strains was found for tetracycline, where 10 (18.9%), 23 (43.4%), and 31 (58.5%) *Salmonella* strains contained the *tet*A, *tet*B, and *tet*C genes, respectively (Table 9). A high percentage of *Salmonella* strains resistant to tetracyclines is consistent with the data from the EFSA and ECDC report [22] from 2022. A relatively high percentage of *Salmonella* strains (35.8%) contained the *sul*1 gene, encoding resistance to sulfamethoxazole, although only two (3.8%) *Salmonella* strains showed phenotypic resistance to sulfamethoxazole with an inhibitor (trimethoprim) (Table 7).

### 3.6. Screening for Phenotypic and Genotypic Detection of β-lactamases-Producing Salmonella Strains

Since the phenotype sensitivity to antibiotics can be conferred by several different antibiotic resistance genes (ARGs), in the last step of our research, the presence of the main mechanisms of β-lactam resistance (phenotypically and genotypically expressed) in *Salmonella* was determined. In *Salmonella*, as in other bacteria from the *Enterobacteriaceae* family, the main mechanism of resistance to β*-*lactam antibiotics are β*-*lactamases encoded by *bla* genes [7,89,90]. Many different β*-*lactamases have been described, but β*-*lactamases of the TEM type (named after the patient Temoneira), CTX*-*M type (active on cefotaxime, first isolated at Munich), and SHV type (sulfhydryl reagent variable) predominate in *Salmonella* [89,91,92,93]. They belong to β*-*lactamases with a broad spectrum of substrate activity (ESBL). ESBL enzymes inactivate cephalosporins and first-, second-, and third-generation penicillins [7,89]. They are not active against carbapenems [94]. ESBL genes of the TEM and CTX*-*M types were not identified among our strains. CTX*-*M enzymes are active against cephalosporins and monobactams and are currently of great epidemiological and clinical importance [7]. The SHV*-*type ESBL gene was identified in two isolates—*S. enterica* strains KKP 1597 and KKP 1610 isolated from food products (Table 4). The presence of the ESBL mechanism was not confirmed phenotypically; therefore, it is likely that the *bla*_SHV_ gene associated with SHV-type ESBL resistance in *S. enterica* KKP 1597 and KKP 1610 strains may be inactive. According to the literature, the presence of *bla*_SHV_ is often associated with the *Enterobacteriaceae* family in nosocomial infections [7]. The presence of *bla*_SHV_ in *Salmonella* strains isolated from hospitalized patients was not confirmed in our study. Another group of β*-*lactamases is AmpC, which confers resistance to all β*-*lactam antibiotics except fourth-generation cephalosporins and carbapenems [95,96]. Contrary to ESBL, the AmpC group is not sensitive to β-lactam inhibitors such as clavulanic acid, sulbactam, and tazobactam [95]. The mechanism of AmpC can be encoded by genes located on chromosomes or plasmids [96]. It has been shown that in *Salmonella*, resistance to broad-spectrum cephalosporins is often associated with *bla*_CMY*–*2_ gene [97]. In our study, none of the strains exhibited a resistance mechanism to AmpC-type β-lactamases. Another gene encoding resistance to β-lactam antibiotics is the *bla*_PSE-1_ gene located on the first-class integron [7,89]. Moreover, the presence of the *bla*_PSE*–*1_ gene associated with the PSE-1 drug efflux mechanism was identified in six *Salmonella* strains, including KKP 1000, KKP 1004, KKP 1005, KKP 1007 isolated from food products, and KKP 3819 and KKP 3820 isolated from poultry. Moreover, no carbapenemase-producing *Salmonella* strains were detected among the tested isolates.

According to the EFSA and ECDC report, the percentage of ESBL and AmpC-producing *Salmonella* strains ranged from very low to low (animal isolates) and very low among isolates obtained from hospitalized patients. Carbapenemase-producing isolates were not detected in any of the zoonotic *Salmonella* strains, while in 2019*–*2020, among isolates from humans, only three carbapenemase-producing *Salmonella* strains were detected [22]. The results from the above-mentioned report are comparable to our study and confirm the low percentage of *Salmonella* strains with resistance mechanisms.

## 4. Conclusions

*Salmonella* isolates show phenotypic resistance to many antibiotics and encode numerous genes associated with antimicrobial resistance. The high number of resistant *Salmonella* strains (isolated both at the end of the 20th century and in recent years) in combination with multiple ARGs indicates the possible irrational/unjustified use of antibiotics for many years. The problem of the development of ESBL or AmpC resistance mechanisms in *Salmonella* strains resulting from both our research and European reports is not alarming yet; however, it is necessary to constantly monitor antimicrobial resistance profiles in all food chain links and to implement a policy of rational antibiotic stewardship (AMS), which may stop or at least significantly limit the further acquisition of antibiotic resistance among *Salmonella* strains. A significant reduction in the use of antibiotics in animal husbandry may limit the transfer of antibiotic resistance genes through food. The development of new, alternative antibacterial agents also represents a relevant approach. One concept that recurs due to the growth of MDR strains is the use of strictly lytic bacteriophages. Currently, phage therapy is an experimental treatment aimed at eradicating bacterial strains for which antibiotic therapy does not bring the expected results. The use of specific bacteriophages in the food industry in the EU countries is not approved for use yet, unlike, for example, in the USA or Canada, where commercial preparations based on phage cocktails against foodborne pathogens for food products are applied.

## Figures and Tables

**Table 1:** The primer pairs used for detection of virulence factors in *Salmonella* strains.

Target Gene	Primer Sequences 5′–3′	Annealing Temperature	Product Size	Reference
*inv*A	F–GTGAAATTATCGCCACGTTCGGGCAA R–TCATCGCACCGTCAAAGGAACC	63 °C	284 bp	[26]
*fim*A	F–CCTTTCTCCATCGTCCTGAA R–TGGTGTTATCTGCCTGACCA	56 °C	85 bp	[27]
*stn*	F–CTTTGGTCGTAAAATAAGGCG R–TGCCCAAAGCAGAGAGATTC	56 °C	260 bp	[28]
*spv*C	F–ACTCCTTGCACAACCAAATGCGGA R–TGTCTTCTGCATTTCGCCACCATCA	63 °C	571 bp	[29]
*spv*R	F–CAGGTTCCTTCAGTATCGCA R–TTTGGCCGGAAATGGTCAGT	56 °C	310 bp	[30]
*rck*	F–CTGACCACCCATTCCGTGT R–GTAACCGACACCAACGTT	56 °C	479 bp	[31]

**Table 2:** The primer pairs and gene targets used for the detection of antimicrobial resistance in *Salmonella* strains.

Target Gene/ Antibiotic	Resistance Mechanism	Primer Sequences 5′-3′	Annealing Temperature	Product Size	Reference
*str*A/*str*B streptomycin	Aminoglicoside phosphotransferase	F–ATGGTGGACCCTAAAACTCT R–CGTCTAGGATCGAGACAAAG	63 °C	891 bp	[35]
*aad*A streptomycin	Streptomycin adenyltransferase	F–GTGGATGGCGGCCTGAAGCC R–AATGCCCAGTCGGCAGCG	63 °C	525 bp	[36]
*aad*B gentamicin	Aminoglycoside transferase	F–GAGGAGTTGGACTATGGATT R–CTTCATCGGCATAGTAAAAG	60 °C	208 bp	[35]
*aac*C gentamicin	Aminoglycoside acetyltransferase	F–GGCGCGATCAACGAATTTATCCGA R–CCATTCGATGCCGAAGGAAACGAT	58 °C	448 bp	[37]
*flo*F florfenicol	Efflux	F–CACGTTGAGCCTCTATATGG R–ATGCAGAAGTAGAACGCGAC	61 °C	888 bp	[7]
*flo*R chloramphenicol	Efflux	F–AACCCGCCCTCTGGATCAAGTCAA R–CAAATCACGGGCCACGCTGTATC	60 °C	548 bp	[38]
*cat*1 chloramphenicol	Chloramphenicol acetyltransferase	F–CCTATAACCAGACCGTTCAG R–TCACAGACGGCATGATGAAC	56 °C	491 bp	[38]
*cat*2 chloramphenicol	Chloramphenicol acetyltransferase	F–CCGGATTGACCTGAATACCT R–TCACATACTGCATGATGAAC	56 °C	456 bp	[38]
*mcr*1 colistin	Phosphoetanolamine transferase	F–AGTCCGTTTGTTCTTGTGGC R–AGATCCTTGGTCTCGGCTTG	58 °C	320 bp	[39]
*mcr*2 colistin	Phosphoetanolamine transferase	F–CAAGTGTGTTGGTCGCAGTT R–TCTAGCCCGACAAGCATACC	58 °C	715 bp	[39]
*mcr*3 colistin	Phosphoetanolamine transferase	F–AAATAAAAATTGTTCCGCTTATG R–AATGGAGATCCCCGTTTTT	58 °C	929 bp	[39]
*mcr*4 colistin	Phosphoetanolamine transferase	F–TCACTTTCATCACTGCGTTG R–TTGGTCCATGACTACCAATG	58 °C	1116 bp	[39]
*mcr*5 colistin	Phosphoetanolamine transferase	F–ATGCGGTTGTCTGCATTTATC R–TCATTGTGGTTGTCCTTTTCTG	58 °C	1644 bp	[39]
*aph*AI-IAB kanamycin	Aminoglycoside phosphoryltranferase	F–AAACGTCTTGCTCGAGGC R–CAAACCGTTATTCATTCGTGA	55 °C	461 bp	[40]
*aph*A1 neomycin	Aminoglicoside phosphotransferase	F–ATGGGCTCGCGATAATGTC R–CTCACCGAGGCAGTTCCAT	60 °C	634 bp	[7]
*aph*A2 neomycin	Aminoglicoside phosphotransferase	F–GATTGAACAAGATGGATTGC R–CCATGATGGATACTTTCTCG	60 °C	347 bp	[7]
*tet*A tetracycline	Efflux	F–GCTACATCCTGCTTGCCTTC R–CATAGATCGCCGTGAAGAGG	56 °C	210 bp	[38]
*tet*B tetracycline	Efflux	F–TTGGTTAGGGGCAAGTTTTG R–GTAATGGGCCAATAACACCG	53 °C	659 bp	[38]
*tet*C tetracycline	Efflux	F–CTTGAGAGCCTTCAACCCAG R–ATGGTCGTCATCTACCTGCC	56 °C	417 bp	[38]
*sul*1 sulfamethoxazole	Dihydropteroate synthase inhibitor	F–CGGCGTGGGCTACCTGAACG R–GCCGATCGCGTGAAGTTCCG	66 °C	433 bp	[35]
*sul*2 sulfamethoxazole	Dihydropteroate synthase inhibitor	F–CGGCATCGTCAACATAACCT R–TGTGCGGATGAAGTCAGCTC	66 °C	721 bp	[35]
*sul*3 sulfamethoxazole	Dihydropteroate synthase inhibitor	F–GGGAGCCGCTTCCAGTAAT R–TCCGTGACACTGCAATCATTA	57 °C	500 bp	[7]
*dfr*A1 trimethoprim	Dihydrofolate reductase	F–CAATGGCTGTTGGTTGGAC R–CCGGCTCGATGTCTATTGT	62 °C	253 bp	[41]
*dfr*A10 trimethoprim	Dihydrofolate reductase	F–TCAAGGCAAATTACCTTGGC R–ATCTATTGGATCACCTACCC	59 °C	433 bp	[41]
*dfr*A12 trimethoprim	Dihydrofolate reductase	F–TTCGCAGACTCACTGAGGG R–CGGTTGAGACAAGCTCGAAT	63 °C	330 bp	[41]

**Table 3:** Primers used for detection of target β-lactamases-related genes in *Salmonella* strains.

Target Gene	Resistance Mechanism	Primer Sequences 5′-3′	Annealing Temperature	Product Size	Reference
*bla*_TEM_	TEM-type ESBL	F–ATGAGTATTCAACATTTCCG R–CTGACAGTTACCAATGCTTA	55 °C	867 bp	[43]
*bla*_CTX-M_	CTX-type ESBL	F–CGCTTTGCGATGTGCAG R–ACCGCGATATCGTTGGT	60 °C	585 bp	[44]
*bla*_SHV_	SHV-type ESBL	F–AGGATTGACTGCCTTTTTG R–ATTTGCTGATTTCGCTCG	55 °C	393 bp	[45]
*bla*_CMY-2_	AmpC	F–GACAGCCTCTTTCTCCACA R–TGGACACGAAGGCTACGTA	55 °C	1000 bp	[45]
*bla*_PSE-1_	Efflux	F–GCAAGTAGGGCAGGCAATCA R–GAGCTAGATAGATGCTCACAA	60 °C	422 bp	[46]

**Table 4:** Source of isolation and taxonomic identification of the *Salmonella* strains.

Bacterial Strain Number	Year of Isolation	Source of Isolation	Bacteria Identification Acc. to MALDI–TOF MS	Bacteria Identification Acc. to *16S* rRNA Sequencing	GenBank Accession Number
KKP 996	1981	HP/fecal sample	*Salmonella enterica* subsp. *enterica*	*Salmonella enterica* subsp. *enterica*	ON627842
KKP 997	1981	FP	*Salmonella enterica* subsp. *enterica*	*Salmonella enterica* subsp. *enterica*	MW046052
KKP 998	1991	FP	*Salmonella enterica* subsp. *enterica*	*Salmonella enterica* subsp. *enterica*	ON764274
KKP 999	1991	FP	*Salmonella enterica* subsp. *enterica*	*Salmonella enterica* subsp. *enterica*	ON627845
KKP 1000	2005	FP	*Salmonella enterica* subsp. *enterica*	*Salmonella enterica* subsp. *enterica*	ON312999
KKP 1001	2005	FP	*Salmonella enterica* subsp. *enterica*	*Salmonella enterica* subsp. *enterica*	MW332255
KKP 1002	2005	FP	*Salmonella* sp.	*Salmonella enterica* subsp. *enterica*	ON340716
KKP 1003	2005	FP	*Salmonella enterica* subsp. *enterica*	*Salmonella enterica* subsp. *enterica*	ON756138
KKP 1004	2005	FP	*Salmonella enterica* subsp. *enterica*	*Salmonella enterica* subsp. *enterica*	ON627844
KKP 1005	2005	FP	*Salmonella enterica* subsp. *enterica*	*Salmonella enterica* subsp. *enterica*	ON627847
KKP 1006	2005	FP	*Salmonella* sp.	*Salmonella enterica* subsp. *enterica*	ON764251
KKP 1007	2005	FP	*Salmonella enterica* subsp. *enterica*	*Salmonella enterica* subsp. *enterica*	ON627846
KKP 1008	2005	FP	*Salmonella enterica* subsp. *enterica*	*Salmonella enterica* subsp. *enterica*	ON340717
KKP 1009	2005	FP	*Salmonella enterica* subsp. *enterica*	*Salmonella enterica* subsp. *enterica*	ON764277
KKP 1010	2005	FP	*Salmonella enterica* subsp. *enterica*	*Salmonella enterica* subsp. *enterica*	ON764279
KKP 1039	2005	FP	*Salmonella enterica* subsp. *enterica*	*Salmonella enterica* subsp. *enterica*	ON764252
KKP 1040	2005	FP	*Salmonella enterica* subsp. *enterica*	*Salmonella enterica* subsp. *enterica*	ON764280
KKP 1041	2005	FP	*Salmonella* sp.	*Salmonella enterica* subsp. *enterica*	ON764253
KKP 1042	2005	FP	*Salmonella enterica* subsp. *enterica*	*Salmonella enterica* subsp. *enterica*	ON798424
KKP 1043	2005	FP	*Salmonella enterica* subsp. *enterica*	*Salmonella enterica* subsp. *enterica*	ON764281
KKP 1044	2005	FP	*Salmonella enterica* subsp. *enterica*	*Salmonella enterica* subsp. *enterica*	ON764287
KKP 1045	2005	FP	*Salmonella enterica* subsp. *enterica*	*Salmonella enterica* subsp. *enterica*	ON764254
KKP 1113	2005	FP/halvah	*Salmonella enterica* subsp. *enterica*	*Salmonella enterica* subsp. *enterica*	ON775567
KKP 1169	2006	FP/sesame seeds	*Salmonella enterica* subsp. *enterica*	*Salmonella enterica* subsp. *enterica*	ON764259
KKP 1193	1987	HP/fecal sample	*Salmonella enterica* subsp. *enterica*	*Salmonella enterica* subsp. *enterica*	ON764258
KKP 1213	2009	FP/caraway seeds	*Salmonella enterica* subsp. *enterica*	*Salmonella enterica* subsp. *enterica*	ON764805
KKP 1217	2009	FP/coriander	*Salmonella enterica* subsp. *enterica*	*Salmonella enterica* subsp. *enterica*	ON764807
KKP 1514	2009	FPL/pump filter	*Salmonella enterica* subsp. *enterica*	*Salmonella enterica* subsp. *enterica*	ON756136
KKP 1597	2009	FP	*Salmonella enterica* subsp. *enterica*	*Salmonella enterica* subsp. *enterica*	ON461374
KKP 1608	2009	FP	*Salmonella enterica* subsp. *enterica*	*Salmonella enterica* subsp. *enterica*	ON312943
KKP 1610	2006	FP	*Salmonella enterica* subsp. *enterica*	*Salmonella enterica* subsp. *enterica*	ON313000
KKP 1611	2009	FP	*Salmonella enterica* subsp. *enterica*	*Salmonella enterica* subsp. *enterica*	ON764857
KKP 1612	2009	FP	*Salmonella enterica* subsp. *enterica*	*Salmonella enterica* subsp. *enterica*	ON764858
KKP 1613	2009	FP	*Salmonella enterica* subsp. *enterica*	*Salmonella enterica* subsp. *enterica*	ON766359
KKP 1614	2009	FP	*Salmonella enterica* subsp. *enterica*	*Salmonella enterica* subsp. *enterica*	ON312941
KKP 1636	2010	FP	*Salmonella enterica* subsp. *enterica*	*Salmonella enterica* subsp. *enterica*	ON773156
KKP 1761	2010	FP	*Salmonella enterica* subsp. *enterica*	*Salmonella enterica* subsp. *enterica*	ON798425
KKP 1762	2010	FP	*Salmonella enterica* subsp. *enterica*	*Salmonella enterica* subsp. *enterica*	ON340720
KKP 1763	2010	FP	*Salmonella enterica* subsp. *enterica*	*Salmonella enterica* subsp. *enterica*	ON773159
KKP 1775	1997	HP/fecal sample	*Salmonella enterica* subsp. *enterica*	*Salmonella enterica* subsp. *enterica*	ON832663
KKP 1776	1995	ABR/poultry	*Salmonella enterica* subsp. *enterica*	*Salmonella enterica* subsp. *enterica*	ON461376
KKP 3078	2019	FP/confectionery industry	*Salmonella enterica* subsp. *enterica*	*Salmonella enterica* subsp. *enterica*	MW034593
KKP 3079	2019	FPL/conveyor belt	*Salmonella enterica* subsp. *enterica*	*Salmonella enterica* subsp. *enterica*	MW033548
KKP 3080	2019	FP/confectionery industry	*Salmonella enterica* subsp. *enterica*	*Salmonella enterica* subsp. *enterica*	MW033536
KKP 3081	2019	FPL/production tank	*Salmonella enterica* subsp. *enterica*	*Salmonella enterica* subsp. *enterica*	MW033602
KKP 3814	2016	ABR/henhouse	*Salmonella enterica* subsp. *enterica*	*Salmonella enterica* subsp. *enterica*	ON732733
KKP 3815	2016	ABR/henhouse	*Salmonella enterica* subsp. *enterica*	*Salmonella enterica* subsp. *enterica*	ON732742
KKP 3816	2016	ABR/henhouse	*Salmonella enterica* subsp. *enterica*	*Salmonella enterica* subsp. *enterica*	ON756119
KKP 3817	2016	ABR/henhouse	*Salmonella enterica* subsp. *enterica*	*Salmonella enterica* subsp. *enterica*	ON756120
KKP 3818	2016	ABR/henhouse	*Salmonella enterica* subsp. *enterica*	*Salmonella enterica* subsp. *enterica*	ON756135
KKP 3819	2018	ABR/poultry	*Salmonella enterica* subsp. *enterica*	*Salmonella enterica* subsp. *enterica*	ON732745
KKP 3820	2018	ABR/poultry	*Salmonella enterica* subsp. *enterica*	*Salmonella enterica* subsp. *enterica*	ON732744
KKP 3821	2018	ABR/poultry	*Salmonella enterica* subsp. *enterica*	*Salmonella enterica* subsp. *enterica*	ON732827

Abbreviations: ABR—animals and animal breeding rooms; FPL—food production lines; FP—food products; HP—hospitalized patients.

**Table 5:** Detection of virulence markers in *Salmonella* strains.

*Salmonella* Strain Number	Virulence Genes					
	*inv*A	*fim*A	*stn*	*spv*C	*spv*R	*rck*
KKP 996	+	+	+	+	+	+
KKP 997	+	+	+	-	−	−
KKP 998	+	+	+	−	−	+
KKP 999	+	+	+	-	-	+
KKP 1000	+	+	+	+	+	+
KKP 1001	+	+	+	−	−	+
KKP 1002	+	+	+	−	−	−
KKP 1003	+	+	+	−	−	−
KKP 1004	+	+	+	−	−	−
KKP 1005	+	+	+	−	−	+
KKP 1006	+	+	+	−	−	−
KKP 1007	+	+	+	−	−	−
KKP 1008	+	+	+	−	−	−
KKP 1009	+	+	+	−	−	−
KKP 1010	+	+	+	−	−	−
KKP 1039	+	+	+	−	−	−
KKP 1040	+	+	+	−	−	−
KKP 1041	+	+	+	−	−	−
KKP 1042	+	+	+	−	−	−
KKP 1043	+	+	+	−	−	−
KKP 1044	+	+	+	−	−	−
KKP 1045	+	+	+	−	−	−
KKP 1113	+	+	+	−	−	−
KKP 1169	+	+	+	−	−	−
KKP 1193	+	+	+	−	−	−
KKP 1213	+	+	+	−	−	+
KKP 1217	+	+	+	−	−	−
KKP 1514	+	+	+	−	−	+
KKP 1597	+	+	+	−	−	−
KKP 1608	+	+	+	−	−	−
KKP 1610	+	+	+	−	−	−
KKP 1611	+	+	+	−	−	−
KKP 1612	+	+	+	−	−	−
KKP 1613	+	+	+	−	−	−
KKP 1614	+	+	+	−	−	−
KKP 1636	+	+	+	+	+	+
KKP 1761	+	+	+	−	−	+
KKP 1762	+	+	+	−	−	−
KKP 1763	+	+	+	−	−	−
KKP 1775	+	+	+	+	+	+
KKP 1776	+	+	+	+	+	+
KKP 3078	+	+	+	+	+	+
KKP 3079	+	+	+	−	−	−
KKP 3080	+	+	+	−	−	−
KKP 3081	+	+	+	−	−	−
KKP 3814	+	+	+	+	+	+
KKP 3815	+	+	+	+	+	+
KKP 3816	+	+	+	+	+	+
KKP 3817	+	+	+	+	+	+
KKP 3818	+	+	+	+	+	+
KKP 3819	+	+	+	+	+	+
KKP 3820	+	+	+	+	+	+
KKP 3821	+	+	+	−	−	−

**Table 6:** Phenotype resistance of *Salmonella* strains.

*Salmonella* Strain Number	Antibiotic Resistance Pattern	MAR Index	MDR
KKP 996	no resistance *	-	
KKP 997	no resistance *	-	
KKP 998	AMC-TTC-FEP-CTX-CPT-CAZ-CT-CRO-ETP-IMP-ATM-PEF-MXF-OFX-AK-CN-TOB	0.61	+
KKP 999	PRL-CPT-CAZ-CRO-PEF-MXF-C	0.25	+
KKP 1000	AMP-SAM-AMC-PRL-TTC-CPT-CN-TOB-C	0.32	+
KKP 1001	CPT-CT-AK-CN-TOB	0.18	
KKP 1002	PRL-CPT-ATM-CIP-PEF-MXF-NOR-CN	0.29	+
KKP 1003	CN-TOB	0.07	
KKP 1004	AMC-TZP-TTC-FEP-CTX-CPT-CT-MXF-OFX-AK-TOB	0.39	+
KKP 1005	CPT-AK	0.07	
KKP 1006	CPT-AK	0.07	
KKP 1007	AMC-TTC-CPT-CT-CRO-MXF-AK-CN-TOB-SXT	0.36	+
KKP 1008	no resistance *	-	
KKP 1009	CPT-CIP-MXF-CN	0.14	+
KKP 1010	CPT-PEF-OFX-AK-SXT	0.18	+
KKP 1039	MXF-AK-TOB	0.11	
KKP 1040	no resistance *	-	
KKP 1041	CPT-AK	0.07	
KKP 1042	CPT	-	
KKP 1043	CPT-ETP-CN-TOB	0.14	+
KKP 1044	AMC-PRL-TZP-TTC-CPT-CRO-IMP-MXF-AK-CN-TOB	0.39	+
KKP 1045	CPT-MXF	0.07	
KKP 1113	AK	-	
KKP 1169	no resistance *	-	
KKP 1193	CT-CN-TOB	0.11	
KKP 1213	PRL-TZP-CPT-CT-CRO-ETP-OFX-AK-CN-TOB	0.36	+
KKP 1217	CPF-CN-TOB	0.11	
KKP 1514	CPT-CT-CRO-ETP-ATM-CIP-MXF-AK-CN-TOB	0.36	+
KKP 1597	CPT-ETP-CIP-MXF-AK-CN-TOB	0.25	+
KKP 1608	no resistance *	-	
KKP 1610	FEP-AK	0.07	
KKP 1611	CPT-AK-TOB	0.11	
KKP 1612	AMC-TTC-CPT-CRO-IMP-PEF-MXF-AK-CN-TOB	0.36	+
KKP 1613	AK	-	
KKP 1614	no resistance *	-	
KKP 1636	PRL-CRO-PEF-MXF-AK-CN-TOB	0.25	+
KKP 1761	CT-CRO-PEF-MXF-NOR-AK-CN-TOB	0.29	+
KKP 1762	AMC-CPT-CT-CRO-MXF-AK-CN-TOB	0.29	+
KKP 1763	CN	-	
KKP 1775	PRL-TZP-FEP-CPT-CT-MXF-CN	0.25	+
KKP 1776	TTC-FEP-AK-TOB	0.14	+
KKP 3078	CPT-MXF-CN	0.11	+
KKP 3079	PRL-CPT-CT-CRO-AK-CN-TOB	0.25	+
KKP 3080	AMC-FEP-CTX-CPT-CT-CRO-MXF-OFX-AK-CN-TOB	0.39	+
KKP 3081	TZP-TTC-FEP-PEF-MXF-AK-CN-TOB	0.29	+
KKP 3814	AK	-	
KKP 3815	AMC-CPT-CT-CRO-CIP-PEF-MXF-AK-CN	0.32	+
KKP 3816	CPT-AK	0.07	
KKP 3817	CPT-ETP-ATM-CIP-MXF-CN	0.21	+
KKP 3818	AK	-	
KKP 3819	PEF	-	
KKP 3820	AMP-SAM-PRL-TTC-CPT-AK-C	0.25	+
KKP 3821	FEP-CTX-CPT-CAZ-CZA-CT-CRO-ETP-ATM-PEF-AK-TOB	0.43	+

* means no resistance to the tested antibiotics. Notes: AMP—ampicillin; SAM—sulbactam/ampicillin; AMC—amoxicillin/clavulanic acid; PRL—piperacillin; TZP—piperacillin/tazobactam; TTC—ticarcillin/clavulanic acid; FEP—cefepime; CTX—cefotaxime; CPT—ceftaroline; CAZ—ceftazidime; CZA—ceftazidime/avibactam; CT—ceftolozane/tazobactam; CRO—ceftriaxone; ETP—ertapenem; IMP—imipenem; ATM—aztreonam; CIP—ciprofloxacin; PEF—pefloxacin; MXF—moxifloxacin; OFX—ofloxacin; NOR—norfloxacin; AK—amikacin; CN—gentamycin; TOB—tobramycin; C—chloramphenicol; SXT—sulphamethoxazole/trimethoprim. Abbreviations: MAR—Multiple Antibiotic Resistance; MDR—Multi-Drug Resistant strain.

**Table 7:** Prevalence of phenotypic antibiotic resistance in *Salmonella* strains.

Antimicrobial Class (*n* = 7)		Antimicrobial Agent (*n* = 28)	Number of Resistant Strains (*n* = 53)	Percentage of Resistant Strains (%)
β–lactam Antibiotics	Penicillins	ampicillin	2	3.8
		sulbactam/ampicillin	2	3.8
		amoxicillin/clavulanic acid	9	17.0
		piperacillin	9	17.0
		piperacillin/tazobactam	5	9.4
		ticarcillin/clavulanic acid	9	17.0
	Cephalosporins	cefepime	8	15.1
		cefotaxime	4	7.6
		ceftaroline	32	60.4
		ceftazidime	3	5.7
		ceftazidime/avibactam	1	1.9
		ceftolozane/tazobactam	14	26.4
		ceftriaxone	14	26.4
	Carbapenems	ertapenem	7	13.2
		imipenem	3	5.7
		meropenem	0	0.0
	Monobactams	aztreonam	5	9.4
Fluoroquinolones		ciprofloxacin	6	11.3
		pefloxacin	11	20.8
		levofloxacin	0	0.0
		moxifloxacin	20	37.7
		ofloxacin	6	11.3
		norfloxacin	2	3.8
Aminoglycosides		amikacin	31	58.5
		gentamycin	26	49.1
		tobramycin	24	45.3
Phenicols		chloramphenicol	3	5.7
Sulfonamides		sulphamethoxazole/trimethoprim	2	3.8

**Table 8:** Distribution of AMR-related genes in relation to antibiotic resistance patterns in *Salmonella* strains.

*Salmonella* Strain Number	Phenotypic Antibiotic Resistance Pattern	Genotypic Antibiotic Resistance Profile
KKP 996	no resistance *	*flo*F, *tet*C
KKP 997	no resistance *	*tet*C
KKP 998	AMC-TTC-FEP-CTX-CPT-CAZ-CT-CRO-ETP-IMP-ATM- PEF-MXF-OFX-AK-CN-TOB	*str*A*/str*B*, flo*F, *aph*A1, *tet*C, *sul*1
KKP 999	PRL-CPT-CAZ-CRO-PEF-MXF-C	*aad*A, *flo*R, *sul*1
KKP 1000	AMP-SAM-AMC-PRL-TTC-CPT-CN-TOB-C	*aad*A, *flo*F, *flo*R, *tet*A, *tet*C, *sul*1
KKP 1001	CPT-CT-AK-CN-TOB	*flo*F, *tet*A
KKP 1002	PRL-CPT-ATM-CIP-PEF-MXF-NOR-CN	*tet*A, *tet*C
KKP 1003	CN-TOB	ND**
KKP 1004	AMC-TZP-TTC-FEP-CTX-CPT-CT-MXF-OFX-AK-TOB	*aad*A, *flo*R, *tet*A, *tet*B, *tet*C, *sul*1
KKP 1005	CPT-AK	*flo*R, *tet*B, *tet*C, *sul*1
KKP 1006	CPT-AK	*tet*C, *sul*1
KKP 1007	AMC-TTC-CPT-CT-CRO-MXF-AK-CN-TOB-SXT	*aad*A, *flo*R, *tet*B, *sul*1
KKP 1008	no resistance *	*tet*B, *tet*C
KKP 1009	CPT-CIP-MXF-CN	*tet*B, *tet*C, *sul*1
KKP 1010	CPT-PEF-OFX-AK-SXT	*str*A/*str*B, *aad*A, *tet*A, *tet*C, *sul*1
KKP 1039	MXF-AK-TOB	*tet*B, *tet*C
KKP 1040	no resistance *	ND**
KKP 1041	CPT-AK	*aad*A, *tet*B, *tet*C, *sul*1
KKP 1042	CPT	*str*A/*str*B, *tet*B, *tet*C, *sul*1
KKP 1043	CPT-ETP-CN-TOB	*tet*C, *sul*1
KKP 1044	AMC-PRL-TZP-TTC-CPT-CRO-IMP-MXF-AK-CN-TOB	*flo*F, *tet*C
KKP 1045	CPT-MXF	*tet*B, *tet*C, *sul*1
KKP 1113	AK	ND**
KKP 1169	no resistance *	*str*A/*str*B, *tet*C, *sul*1, *sul*2
KKP 1193	CT-CN-TOB	*tet*C, *sul*1
KKP 1213	PRL-TZP-CPT-CT-CRO-ETP-OFX-AK-CN-TOB	*tet*B
KKP 1217	CPF-CN-TOB	*tet*B, *sul*1
KKP 1514	CPT-CT-CRO-ETP-ATM-CIP-MXF-AK-CN-TOB	*tet*B, *tet*C
KKP 1597	CPT-ETP-CIP-MXF-AK-CN-TOB	*tet*A, *tet*B
KKP 1608	no resistance *	*flo*F
KKP 1610	FEP-AK	*sul*1
KKP 1611	CPT-AK-TOB	ND**
KKP 1612	AMC-TTC-CPT-CRO-IMP-PEF-MXF-AK-CN-TOB	*tet*C
KKP 1613	AK	*tet*C
KKP 1614	no resistance *	*tet*C
KKP 1636	PRL-CRO-PEF-MXF-AK-CN-TOB	*tet*A, *tet*B, *tet*C
KKP 1761	CT-CRO-PEF-MXF-NOR-AK-CN-TOB	*tet*B, *tet*C
KKP 1762	AMC-CPT-CT-CRO-MXF-AK-CN-TOB	*tet*B
KKP 1763	CN	*tet*B, *tet*C
KKP 1775	PRL-TZP-FEP-CPT-CT-MXF-CN	ND**
KKP 1776	TTC-FEP-AK-TOB	*tet*C
KKP 3078	CPT-MXF-CN	*tet*B
KKP 3079	PRL-CPT-CT-CRO-AK-CN-TOB	*tet*A, *tet*C
KKP 3080	AMC-FEP-CTX-CPT-CT-CRO-MXF-OFX-AK-CN-TOB	*flo*F, *tet*A, *tet*C
KKP 3081	TZP-TTC-FEP-PEF-MXF-AK-CN-TOB	*tet*A, *tet*B, *tet*C
KKP 3814	AK	*tet*B
KKP 3815	AMC-CPT-CT-CRO-CIP-PEF-MXF-AK-CN	*tet*B
KKP 3816	CPT-AK	ND**
KKP 3817	CPT-ETP-ATM-CIP-MXF-CN	*tet*B
KKP 3818	AK	*tet*B
KKP 3819	PEF	*flo*R, *tet*C, *sul*1
KKP 3820	AMP-SAM-PRL-TTC-CPT-AK-C	*aad*A, *flo*R, *aph*AI-IAB, *sul*1
KKP 3821	FEP-CTX-CPT-CAZ-CZA-CT-CRO-ETP-ATM-PEF-AK-TOB	ND **

* means no resistance to the tested antibiotics | ** ND means: no resistance genes were detected. **Notes:** AMP—ampicillin; SAM—sulbactam/ampicillin; AMC—amoxicillin/clavulanic acid; PRL—piperacillin; TZP—piperacillin/tazobactam; TTC—ticarcillin/clavulanic acid; FEP—cefepime; CTX—cefotaxime; CPT—ceftaroline; CAZ—ceftazidime; CZA—ceftazidime/avibactam; CT—ceftolozane/tazobactam; CRO—ceftriaxone; ETP—ertapenem; IMP—imipenem; ATM—aztreonam; CIP–ciprofloxacin; PEF—pefloxacin; MXF—moxifloxacin; OFX—ofloxacin; NOR—norfloxacin; AK—amikacin; CN—gentamycin; TOB—tobramycin; C—chloramphenicol; SXT—sulphamethoxazole/trimethoprim

**Table 9:** Prevalence of genotypic antibiotic resistance in *Salmonella* strains.

Antibiotic	Target Gene	Number of Resistant Strains (*n* = 53)	Percentage of Resistant Strains (%)
streptomycin	*str*A/*str*B	4	7.6
	*aad*A	7	13.2
florfenicol	*flo*F	7	13.2
chloramphenicol	*flo*R	7	13.2
kanamycin	*aph*AI-IAB	1	1.9
neomycin	*aph*A1	1	1.9
tetracycline	*tet*A	10	18.9
	*tet*B	23	43.4
	*tet*C	31	58.5
sulfamethoxazole	*sul*1	19	35.8
	*sul*2	1	1.9

## Data Availability

Not applicable.

## Supplementary Materials

The following are available online at https://www.mdpi.com/article/10.3390/pathogens11111323/s1, Table S1: The PCR conditions for amplification of virulence markers, AMR-related and β-lactamases-related genes in *Salmonella* strains.
