# Supplementary material for: Bacterial Pathogens in the Food Industry: Antibiotic Resistance and Virulence Factors of Salmonella enterica Strains Isolated from Food Chain Links
Source: Pathogens. 2022 Nov 10;11(11):1323. doi: 10.3390/pathogens11111323 (PMC9692263; doi:10.3390/pathogens11111323)
Supplement: Supplementary file 1 [file pathogens-11-01323-s001.zip › pathogens-2009883-supplementary.pdf]

Table S1. The PCR conditions for amplification of virulence markers, AMR–related and β–lactamases–related genes in *Salmonella* strains.

Target Gene	Primer Sequences 5′–3′	PCR conditions	Product Size
*inv*A	F–GTGAAATTATCGCCACGTTCGGGCAA<br>R–TCATCGCACCGTCAAAGGAACC	initial denaturation step of 5 min at 94 °C, followed by 35 cycles as follows: 30 sec at 94 °C for denaturation, 30 sec at 63 °C for annealing, 30 sec at 72°C for extension and a final extension step of 10 min at 72 °C	284 bp
*fim*A	F–CCTTTCTCCATCGTCCTGAA<br>R–TGGTGTTATCTGCCTGACCA	initial denaturation step of 5 min at 94 °C, followed by 35 cycles as follows: 30 sec at 94 °C for denaturation, 30 sec at 56 °C for annealing, 30 sec at 72°C for extension and a final extension step of 10 min at 72 °C	85 bp
stn	F–CTTTGGTCGTAAAATAAGGCG<br>R–TGCCCAAAGCAGAGAGATTC	initial denaturation step of 5 min at 94 °C, followed by 35 cycles as follows: 30 sec at 94 °C for denaturation, 30 sec at 56 °C for annealing, 30 sec at 72°C for extension and a final extension step of 10 min at 72 °C	260 bp
*spv*C	F–ACTCCTTGCACAACCAAATGCGGA<br>R–TGTCTTCTGCATTTCGCCACCATCA	initial denaturation step of 5 min at 94 °C, followed by 35 cycles as follows: 30 sec at 94 °C for denaturation, 30 sec at 63 °C for annealing, 30 sec at 72°C for extension and a final extension step of 10 min at 72 °C	571 bp
*spv*R	F–CAGGTTCCTTCAGTATCGCA<br>R–TTTGGCCGGAAATGGTCAGT	initial denaturation step of 5 min at 94 °C, followed by 35 cycles as follows: 30 sec at 94 °C for denaturation, 30 sec at 56 °C for annealing, 30 sec at 72°C for extension and a final extension step of 10 min at 72 °C	310 bp
rck	F–CTGACCACCCATTCCGTGT<br>R–GTAACCGACACCAACGTT	initial denaturation step of 5 min at 94 °C, followed by 35 cycles as follows: 30 sec at 94 °C for denaturation, 30 sec at 56 °C for annealing, 30 sec at 72°C for extension and a final extension step of 10 min at 72 °C	479 bp
*str*A/*str*B	F–ATGGTGGACCCTAAAACTCT<br>R–CGTCTAGGATCGAGACAAAG	initial denaturation step of 10 min at 94 °C, followed by 30 cycles as follows: 30 sec at 94 °C for denaturation, 1 min at 63 °C for annealing, 30 sec at 72°C for extension and a final extension step of 10 min at 72 °C	891 bp
*aad*A	F–GTGGATGGCGGCCTGAAGCC<br>R–AATGCCCAGTCGGCAGCG	initial denaturation step of 10 min at 94 °C, followed by 30 cycles as follows: 30 sec at 94 °C for denaturation, 1 min at 63 °C for annealing, 30 sec at 72°C for extension and a final extension step of 10 min at 72 °C	525 bp
*aad*B	F–GAGGAGTTGGACTATGGATT<br>R–CTTCATCGGCATAGTAAAAG	initial denaturation step of 7 min at 95 °C, followed by 35 cycles as follows: 30 sec at 94 °C for denaturation, 30 sec at 60 °C for annealing, 45 sec at 72°C for extension and a final extension step of 10 min at 72 °C	208 bp
*aac*C	F–GGCGCGATCAACGAATTTATCCGA<br>R–CCATTCGATGCCGAAGGAAACGAT	initial denaturation step of 5 min at 95 °C, followed by 30 cycles as follows: 1 min at 95 °C for denaturation, 1 min at 58 °C for annealing, 1 min at 72°C for extension and a final extension step of 7 min at 72 °C	448 bp
*flo*F	F–CACGTTGAGCCTCTATATGG<br>R–ATGCAGAAGTAGAACGCGAC	initial denaturation step of 10 min at 94 °C, followed by 30 cycles as follows: 1 min at 94 °C for denaturation, 1 min at 61 °C for annealing, 1 min at 72°C for extension and a final extension step of 10 min at 72 °C	888 bp

Table S1. *Cont.*

Target Gene	Primer Sequences 5′–3′	PCR conditions	Product Size
*flo*R	F–AACCCGCCCTCTGGATCAAGTCAA<br>R–CAAATCACGGGCCACGCTGTATC	initial denaturation step of 10 min at 94 °C, followed by 30 cycles as follows: 1 min at 94 °C for denaturation, 1 min at 60 °C for annealing, 3 min at 72°C for extension and a final extension step of 10 min at 72 °C	548 bp
*cat*1	F–CCTATAACCAGACCGTTCAG<br>R–TCACAGACGGCATGATGAAC	initial denaturation step of 5 min at 94 °C, followed by 30 cycles as follows: 1 min at 94 °C for denaturation, 1 min at 56 °C for annealing, 1 min at 72°C for extension and a final extension step of 10 min at 72 °C	491 bp
*cat*2	F–CCGGATTGACCTGAATACCT<br>R–TCACATACTGCATGATGAAC	initial denaturation step of 5 min at 94 °C, followed by 30 cycles as follows: 1 min at 94 °C for denaturation, 1 min at 56 °C for annealing, 1 min at 72°C for extension and a final extension step of 10 min at 72 °C	456 bp
*mcr*1	F–AGTCCGTTTGTTCTTGTGGC<br>R–AGATCCTTGGTCTCGGCTTG	initial denaturation step of 15 min at 94 °C, followed by 35 cycles as follows: 30 sec at 94 °C for denaturation, 90 sec at 58 °C for annealing, 1 min at 72°C for extension and a final extension step of 10 min at 72 °C	320 bp
*mcr*2	F–CAAGTGTGTTGGTCGCAGTT<br>R–TCTAGCCCGACAAGCATACC	initial denaturation step of 15 min at 94 °C, followed by 35 cycles as follows: 30 sec at 94 °C for denaturation, 90 sec at 58 °C for annealing, 1 min at 72°C for extension and a final extension step of 10 min at 72 °C	715 bp
*mcr*3	F–AAATAAAAATTGTTCCGCTTATG<br>R–AATGGAGATCCCCGTTTTT	initial denaturation step of 15 min at 94 °C, followed by 35 cycles as follows: 30 sec at 94 °C for denaturation, 90 sec at 58 °C for annealing, 1 min at 72°C for extension and a final extension step of 10 min at 72 °C	929 bp
*mcr*4	F–TCACTTTCATCACTGCGTTG<br>R–TTGGTCCATGACTACCAATG	initial denaturation step of 15 min at 94 °C, followed by 35 cycles as follows: 30 sec at 94 °C for denaturation, 90 sec at 58 °C for annealing, 1 min at 72°C for extension and a final extension step of 10 min at 72 °C	1116 bp
*mcr*5	F–ATGCGGTTGTCTGCATTTATC<br>R–TCATTGTGGTTGTCCTTTTCTG	initial denaturation step of 15 min at 94 °C, followed by 35 cycles as follows: 30 sec at 94 °C for denaturation, 90 sec at 58 °C for annealing, 1 min at 72°C for extension and a final extension step of 10 min at 72 °C	1644 bp
*aph*AI-IAB	F–AAACGTCTTGCTCGAGGC<br>R–CAAACCGTTATTCATTCGTGA	initial denaturation step of 5 min at 95 °C, followed by 30 cycles as follows: 1 min at 95 °C for denaturation, 1 min at 55 °C for annealing, 1 min at 72°C for extension and a final extension step of 7 min at 72 °C	461 bp
*aph*A1	F–ATGGGCTCGCGATAATGTC<br>R–CTCACCGAGGCAGTTCCAT	initial denaturation step of 7 min at 95 °C, followed by 35 cycles as follows: 30 sec at 94 °C for denaturation, 30 sec at 60 °C for annealing, 45 sec at 72°C for extension and a final extension step of 10 min at 72 °C	634 bp

Table S1. *Cont.*

Target Gene	Primer Sequences 5′–3′	PCR conditions	Product Size
*aph*A2	F–GATTGAACAAGATGGATTGC<br>R–CCATGATGGATACTTTCTCG	initial denaturation step of 7 min at 95 °C, followed by 35 cycles as follows: 30 sec at 94 °C for denaturation, 30 sec at 60 °C for annealing, 45 sec at 72°C for extension and a final extension step of 10 min at 72 °C	347 bp
*tet*A	F–GCTACATCCTGCTTGCCTTC<br>R–CATAGATCGCCGTGAAGAGG	initial denaturation step of 5 min at 94 °C, followed by 35 cycles as follows: 1 min at 94 °C for denaturation, 1 min at 56 °C for annealing, 1 min at 72°C for extension and a final extension step of 7 min at 72 °C	210 bp
*tet*B	F–TTGGTTAGGGGCAAGTTTTG<br>R–GTAATGGGCCAATAACACCG	initial denaturation step of 5 min at 94 °C, followed by 35 cycles as follows: 1 min at 94 °C for denaturation, 1 min at 53 °C for annealing, 1 min at 72°C for extension and a final extension step of 7 min at 72 °C	659 bp
*tet*C	F–CTTGAGAGCCTTCAACCCAG<br>R–ATGGTCGTCATCTACCTGCC	initial denaturation step of 5 min at 94 °C, followed by 35 cycles as follows: 1 min at 94 °C for denaturation, 1 min at 56 °C for annealing, 1 min at 72°C for extension and a final extension step of 7 min at 72 °C	417 bp
*sul*1	F–CGGCGTGGGCTACCTGAACG<br>R–GCCGATCGCGTGAAGTTCCG	initial denaturation step of 10 min at 94 °C, followed by 30 cycles as follows: 1 min at 94 °C for denaturation, 1 min at 66 °C for annealing, 1 min at 72°C for extension and a final extension step of 10 min at 72 °C	433 bp
*sul*2	F–CGGCATCGTCAACATAACCT<br>R–TGTGCGGATGAAGTCAGCTC	initial denaturation step of 10 min at 94 °C, followed by 30 cycles as follows: 1 min at 94 °C for denaturation, 1 min at 66 °C for annealing, 1 min at 72°C for extension and a final extension step of 10 min at 72 °C	721 bp
*sul*3	F–GGGAGCCGCTTCCAGTAAT<br>R–TCCGTGACACTGCAATCATTA	initial denaturation step of 5 min at 94 °C, followed by 30 cycles as follows: 45 sec at 94 °C for denaturation, 45 sec at 57 °C for annealing, 45 sec at 72°C for extension and a final extension step of 10 min at 72 °C	500 bp
*dfr*A1	F–CAATGGCTGTTGGTTGGAC<br>R–CCGGCTCGATGTCTATTGT	initial denaturation step of 5 min at 94 °C, followed by 30 cycles as follows: 45 sec at 94 °C for denaturation, 45 sec at 62 °C for annealing, 45 sec at 72°C for extension and a final extension step of 10 min at 72 °C	253 bp
*dfr*A10	F–TCAAGGCAAATTACCTTGGC<br>R–ATCTATTGGATCACCTACCC	initial denaturation step of 5 min at 94 °C, followed by 30 cycles as follows: 45 sec at 94 °C for denaturation, 45 sec at 59 °C for annealing, 45 sec at 72°C for extension and a final extension step of 10 min at 72 °C	433 bp
*dfr*A12	F–TTCGCAGACTCACTGAGGG<br>R–CGGTTGAGACAAGCTCGAAT	initial denaturation step of 5 min at 94 °C, followed by 30 cycles as follows: 45 sec at 94 °C for denaturation, 45 sec at 63 °C for annealing, 45 sec at 72°C for extension and a final extension step of 10 min at 72 °C	330 bp

Table S1. *Cont.*

Target Gene	Primer Sequences 5′–3′	PCR conditions	Product Size
bla$_{\text{TEM}}$	F–ATGAGTATTCAACATTTCCG<br>R–CTGACAGTTACCAATGCTTA	initial denaturation step of 5 min at 95 °C, followed by 35 cycles as follows: 1 min at 95 °C for denaturation, 1 min at 55 °C for annealing, 1 min at 72°C for extension and a final extension step of 7 min at 72 °C	867 bp
bla$_{\text{CTX-M}}$	F–CGCTTTGCGATGTGCAG<br>R–ACCGCGATATCGTTGGT	initial denaturation step of 5 min at 95 °C, followed by 35 cycles as follows: 1 min at 95 °C for denaturation, 1 min at 60 °C for annealing, 1 min at 72°C for extension and a final extension step of 10 min at 72 °C	585 bp
bla$_{\text{SHV}}$	F–AGGATTGACTGCCTTTTTG<br>R–ATTTGCTGATTTCGCTCG	initial denaturation step of 5 min at 94 °C, followed by 30 cycles as follows: 1 min at 94 °C for denaturation, 1 min at 55 °C for annealing, 1 min at 72°C for extension and a final extension step of 10 min at 72 °C	393 bp
bla$_{CMY\text{-}2}$	F–GACAGCCTCTTTCTCCACA<br>R–TGGACACGAAGGCTACGTA	initial denaturation step of 5 min at 94 °C, followed by 30 cycles as follows: 1 min at 94 °C for denaturation, 1 min at 55 °C for annealing, 1 min at 72°C for extension and a final extension step of 10 min at 72 °C	1000 bp
bla$_{PSE\text{-}1}$	F–GCAAGTAGGGCAGGCAATCA<br>R–GAGCTAGATAGATGCTCACAA	initial denaturation step of 5 min at 95 °C, followed by 30 cycles as follows: 45 sec at 95 °C for denaturation, 45 sec at 60 °C for annealing, 45 sec at 72°C for extension and a final extension step of 10 min at 72 °C	422 bp
